# Patient Perspectives on Open-Door Policies in Psychiatry: Mixed Methods Study

**DOI:** 10.2196/73610

**Published:** 2025-08-08

**Authors:** Timur Liwinski, Robert Davidson, Jan Sarlon, Rainer Gaupp, Lukas Imfeld, Annette B Brühl, Marc Vogel, Christian G Huber, Undine E Lang

**Affiliations:** 1 Clinic for Adults, Universitäre Psychiatrische Kliniken Basel, Wilhelm-Klein-Strasse 27, Basel, 4002, Switzerland, 41 782050661

**Keywords:** open-door policy, psychiatric care, autonomy, patient perspectives, text mining, coercion, ward environment

## Abstract

**Background:**

Open-door policies in psychiatric wards are increasingly recommended as a means to reduce coercion and enhance patient autonomy. However, evidence that integrates patient perspectives on ward openness and related safety measures remains limited. Traditional qualitative approaches often lack the breadth to fully capture the complexity of these views. We hypothesized that patients would prefer open-door treatment and hold a critical view of locked-ward environments, emphasizing autonomy and dignity in care.

**Objective:**

This study aims to systematically explore psychiatric patients’ perspectives on open-door versus locked-ward treatment, identifying key themes and quantifying preferences within a large clinical sample.

**Methods:**

A hybrid questionnaire survey was conducted in September 2023 at the University Psychiatric Clinics (UPK) Basel. The survey examined psychiatric service usage and integrated key factors from a meta-review, including ward relationships, environment, autonomy, legal status, coercion, care entitlement, and expectations at admission and discharge. The final sample comprised 604 patients (response rate 19.1%) drawn from an initial pool of 3212 former inpatients. A text mining approach using latent Dirichlet allocation, a Bayesian topic modeling technique, was applied to analyze open-ended responses and identify latent thematic structures.

**Results:**

The majority of respondents (347/544, 63.8%) rated open-door treatment as “very important” (10 out of 10 on a Likert scale). In contrast, only 21.0% (127/552) of participants were willing to accept voluntary treatment in locked wards, with 70.4% (425/552) explicitly rejecting this option. Logistic regression indicated that younger patients were significantly more likely to accept locked ward treatment (β=−.18, *P*=.04), while patients diagnosed with mood disorders (*ICD-10* [*International Statistical Classification of Diseases and Related Health Problems, Tenth Revision*] F3) showed a trend toward lower acceptance (β=−.42, *P*=.08). Gender and other diagnoses were not significant predictors. Latent Dirichlet allocation identified 5 key topics within patient narratives, which hierarchical clustering grouped into 2 overarching themes: Restriction and Institutionalization, characterized by terms indicating confinement, loss of control, and social isolation; and Autonomy and Self-Determination, which emphasizes patients’ desire for freedom, control over daily life, and access to nature and outdoor spaces.

**Conclusions:**

This study provides robust evidence that psychiatric patients overwhelmingly prioritize open-door policies, linking them to enhanced autonomy, trust, and therapeutic engagement. The thematic analysis highlights the psychological and social costs of locked wards and the critical need for flexible, patient-centered care models. Younger age and diagnostic category influence willingness to accept locked settings, suggesting the need for tailored approaches. Institutions aiming to implement open-door policies should consider these preferences alongside adequate staffing, therapeutic programming, and environmental modifications to foster autonomy while maintaining safety. Integrating patient perspectives in policy design may enhance treatment satisfaction and clinical outcomes.

## Introduction

Medicine has advanced greatly over the years, but the 4 core ethical principles—beneficence, nonmaleficence, justice, and autonomy—remain a constant priority in clinical practice [1]. Approximately 6%‐8% of patients in psychiatric hospitals are subjected to coercive measures involving restriction or deprivation of liberty at some point during their inpatient stay. In addition, roughly 1 in 200 patients undergoes involuntary medication [2]. Although coercive measures affect only a minority of patients in psychiatric hospitals, they represent a serious challenge to psychiatry’s core ethical principles [3]. In Switzerland, the introduction of the revised Child and Adult Protection Law in 2013 was accompanied by the hope of reducing coercive measures in psychiatry. However, this expectation has not been fulfilled. On the contrary, the number of coercive interventions—such as involuntary hospitalizations and liberty-restricting measures—has increased. According to the Swiss Health Observatory (Obsan), 18,367 adults were admitted to psychiatric hospitals without consent in 2022, accounting for over a quarter of all psychiatric inpatients [4], in a country with a population of approximately 8.8 million at that time [5]. The management of psychiatric inpatients varies widely across European health care systems, with containment strategies and policies for handling violence often shaped more by local tradition than by empirical evidence [6-8]. Nonpharmacological interventions for disruptive or aggressive behavior remain largely unsupported by controlled studies [7], and the use of locked wards continues to be a contentious issue, marked by significant institutional and regional differences [8]. Locked wards are frequently described as shifting from therapeutic care to custodial containment, with restrictive policies being linked to increased incidents of aggression, self-harm, and coercive interventions, potentially reinforcing the very behaviors they aim to prevent [9-17]. The physical and procedural constraints of these settings—locked doors, limited access to therapeutic spaces, and rigid institutional routines—have been reported to compromise privacy, exacerbate frustration, and reduce opportunities for meaningful engagement in care [18-23]. Amid the growing emphasis within psychiatry on patient autonomy and self-determination, the term “open door policy” has gained increasing prominence in both clinical initiatives and the scientific literature. Despite the lack of a universally endorsed definition by major professional societies, we adopt the definition provided by the Council of Europe’s Section on Human Rights and Biomedicine, which characterizes an “open door policy” as “a policy of maintaining open doors in mental health settings and particularly hospital-based settings that otherwise would be ‘closed’ or ‘locked’.” This approach aims to support patient autonomy and mitigate the detrimental effects of institutionalization associated with closed settings [24]. In our view, this definition is largely consistent with the term’s usage in high-impact medical publications in the field [25-31].

Despite the central role that patient experiences play in shaping psychiatric care, empirical research capturing their perspectives remains scarce [32,33]. Understanding how inpatients perceive locked environments is essential for informing policies that balance safety with autonomy. In this study, we conducted a survey at the University Psychiatric Clinics (Universitäre Psychiatrische Kliniken; UPK) Basel, Switzerland, to explore patients’ views on open-door policies, accessibility to therapy spaces, and interactions with clinical staff. This initiative was undertaken as part of a feasibility assessment for a new construction project within the Adult Psychiatry Department, prompting a revision of its operational concept.

The usage of textual datasets is growing in the psychological sciences since they may offer significant insights on patterns and correlations pertaining to human behavior and attitudes [34]. To systematically analyze patient narratives, we used topic modeling, a machine learning approach that identifies latent themes in large textual datasets. Unlike traditional qualitative methods, which are labor-intensive and limited in scope, text mining using probabilistic topic models allows for the efficient processing of extensive free-text responses [35]. Through this approach, we aimed to derive a data-driven understanding of patient perspectives on locked wards, providing empirical insights that could inform psychiatric care practices and institutional policy. Based on the existing literature and the growing emphasis on patient-centered care, we hypothesized that patients would express a preference for open-door policies and a more apprehensive stance toward closed-door wards, emphasizing the significance of autonomy and dignity in their care.

## Methods

### Psychiatric Facility

The UPK at the University of Basel, Switzerland, provides psychiatric services to both inpatient and outpatient populations in the city of Basel and its surrounding regions, serving approximately 190,000 residents. Over the study duration, the clinic for adults consistently maintained a bed capacity of 277 for inpatient care. Our center adopts a “Track Units” organizational model, structured in a decentralized and modular manner based on symptoms and syndromes. This design facilitates tailored treatment approaches that address the unique stage-specific requirements of patients across both inpatient and outpatient sectors [36]. As of December 31, 2023, the UPK Basel used 1200 staff members (previous year: 1207 staff members). In recent years, bed occupancy remained stable at approximately 92%, with an average length of stay of 37.8 days in 2023 [37].

### Sample Size Considerations

In 2023, a total of 3120 inpatient cases were treated at the UPK Basel. Based on historical data indicating a response rate of just under 20% for similar surveys in this patient population, we anticipated a comparable return rate. To ensure sufficient statistical power even under an unlikely and pessimistic response scenario, we selected a stratified random sample of >3000 former patients. This approach exceeded the minimum required sample size as calculated using the Cochran formula [38], which indicates that a sample of 246 individuals is sufficient to estimate the population proportion with a 5% margin of error at a 95% confidence level, assuming a response rate of approximately 20%. Ultimately, we obtained 604 usable responses, providing a robust basis for analysis. It has previously been demonstrated that, even in cases of extremely low response rates, survey results can remain largely unbiased, provided the sample size is sufficiently large. Response rates as low as 4% have been shown to yield reliable estimates under such conditions [39].

We aimed to ensure a sufficiently large sample size to support the reliability of topic modeling results. While there is no established consensus on the optimal number of documents required for latent Dirichlet allocation (LDA), a recent systematic review in psychological science found that only 35% of studies reported sample sizes, which varied widely—from 69 to over 114 million documents. Our dataset falls well within this observed range, suggesting adequacy for exploratory topic modeling in this context.

### Questionnaire-Derived Data

This analysis is based on a hybrid questionnaire survey conducted in September 2023 at the UPK, examining patient preferences for psychiatric inpatient settings (Multimedia Appendix 1). The survey incorporated key factors from a meta-review, including ward relationships, environment, autonomy, legal status, coercion, perceived care entitlement, and expectations at admission and discharge [40]. An interdisciplinary team of physicians, nurses, psychologists, former patients and peers, quality experts, and a hospital architecture specialist developed the questionnaire. It covered 4 key areas—person, room, ward, and clinic—using Likert scales, multiple-choice, and binary questions to assess patient needs and preferences, supplemented by open-ended questions for deeper insights. A stratified random sample, encompassing 3212 individuals who had undergone clinic experiences from April 2021 to August 2023, was drawn from the patient registry of the hospital. The survey instruments were disseminated among the selected individuals, with electronic invitations sent to those possessing an accessible email address (44.8%), and paper questionnaires dispatched, along with prepaid return envelopes, to individuals without email access. The questionnaire was administered using the web-based Enterprise Feedback Suite by Tivian [41]. Survey data collection continued until the end of October, with student assistants using Remark Office Optical Mark Recognition for extracting data from the paper questionnaires [42].

### Text Mining Approach

Analyzing large collections of written responses poses significant challenges when done manually, due to the time and effort required. To address this, we applied a widely used computational method called topic modeling, which helps uncover recurring themes in text data by identifying patterns of word usage across documents. Specifically, we used LDA—a type of machine learning algorithm often applied in health research. This method groups words that frequently appear together [35] and organizes them into “topics” that reflect underlying themes in the text [43]. It estimates the likelihood of certain topics appearing in each response and the likelihood of specific words being associated with each topic, allowing us to explore the latent structure of patient narratives.

### Data Analysis

All analyses were conducted using R (version 4.4.0; R Core Team). We conducted an exploratory binary logistic regression analysis to examine predictors of patients’ willingness to voluntarily enter a locked psychiatric ward. The outcome variable was voluntary admission to a closed ward. Predictor variables included age group (numerical), gender, and diagnostic categories: severe mental illness, psychotic disorders (F20-F29), affective disorders (F30-F39), substance use disorders (F10-F19), and geriatric psychiatry. The logistic regression model was fitted using the “glm()” function in R (family=binomial). Model assumptions and diagnostics were checked using the “performance” package.

Text processing and cleaning included standard procedures such as converting text to lowercase, removing punctuation, numbers, and common stopwords (eg, “and,” “the”), and consolidating similar words (eg, singular or plural forms). A document-term matrix was then created to quantify how often words appeared across responses. LDA modeling was performed using the topicmodels package [44], and the number of topics was guided by model performance metrics (eg, perplexity) and interpretability. To better understand how topics relate to one another, we used hierarchical clustering using “ward.D2” agglomeration method based on shared words across topics. This allowed us to group similar themes and present a clearer picture of the main concerns expressed by participants. Our final number of topics was chosen based on both algorithmic output and expert judgment, balancing statistical rigor with clinical interpretability. Two independent reviewers (TL and RD) analyzed the extracted topics, reviewed the top terms and representative texts, and resolved any discrepancies through discussion to ensure consistent and meaningful interpretation. To complement these analyses and provide a visual representation of key themes, we created node diagrams illustrating the term co-occurrence network within topics. Terms were connected if they appeared together within the same topic, and the frequency of co-occurrence determined the edge weight. The network graph was constructed using the igraph, tidygraph, and ggraph packages in R, with layout and aesthetics optimized for clarity and interpretability. This visualization highlights the core lexical structure of each topic and the interrelations between prominent terms.

### Ethical Considerations

This study was conducted as part of a clinical quality development project aimed at informing the design of future psychiatric clinic infrastructure. As such, the initial patient survey and questionnaire development were implemented within the framework of routine clinical quality assurance and not as a research study involving human participants. Therefore, formal ethical approval was not required at that stage. For this study, all analyses were conducted using retrospectively collected, fully anonymized secondary data, with no identifiable personal or health information involved. In line with national regulations, this type of research is exempt from formal ethics committee approval. A nonresponsibility declaration was obtained from the Ethics Committee Northwest and Central Switzerland, confirming that the study did not fall under the Human Research Act and did not require ethics approval (Req-2024-00502). Participation in the original survey was voluntary, and patients were informed about the purpose of the quality development initiative. By completing the survey, they provided implied informed consent. No financial or material compensation was provided to participants. All procedures were conducted in accordance with relevant data protection and privacy regulations.

## Results

### Quantitative Survey Findings

Among the 3212 former patients initially invited (Table 1), the majority were most recently treated for mood (affective) disorders (1035/3212, 32.22%; *ICD-10* [*International Statistical Classification of Diseases and Related Health Problems, Tenth Revision*] F3), followed by mental and behavioral disorders due to psychoactive substance use (626/3212, 19.49%; *ICD-10* F1). The final sample comprised 604 individuals, yielding a response rate of 19.1%. Overall, 55.0% (332/604) of the participants in the study self-identified as female. The age distribution of participants spanned from 16‐25 years (73/604, 12.1%), 26‐40 years (162/604, 26.9%), 41‐55 years (172/604, 28.4%), 56‐65 years (123/604, 20.3%), 66‐70 years (28/604, 4.6%), to more than 70 years (47/604, 7.7%), with the largest proportion in the 41‐55 age group.

**Table 1.** Diagnostic distribution of invited inpatients (N=3212).

*ICD-10^a^* code	Diagnostic category	Absolute frequency, n	Relative frequency, %
F0	Organic mental disorders (eg, dementia)	40	1.25
F1	Mental and behavioral disorders due to psychoactive substance use	626	19.49
F2	Schizophrenia, schizotypal, and delusional disorders	430	13.39
F3	Mood (affective) disorders	1035	32.22
F4	Neurotic, stress-related, and somatoform disorders	578	18.00
F6	Disorders of adult personality and behavior	415	12.92
Other	Other mental disorders	88	2.74

a *ICD-10*: *International Statistical Classification of Diseases and Related Health Problems, Tenth Revision*.

**Figure 1.**
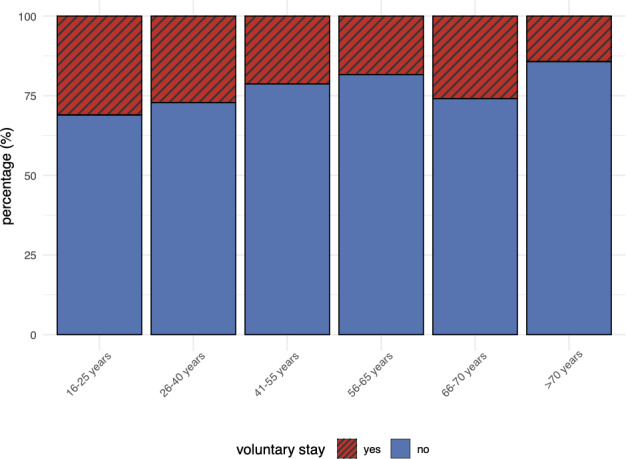
Bar chart showing the age distribution stratified by willingness to be voluntarily treated in a locked ward (yes vs no).

A significant majority of respondents highlighted the importance of open-door treatment in psychiatric care, with approximately two-thirds (347/544, 63.8%) rating it as “very important” (10 out of 10 on a Likert scale). With a further 9.6% (52/544) of respondents rating it 9 out of 10 and 13.2% (72/544) assigning it a score of 8 out of 10, only 0.9% (5/544) considered it unimportant, rating it 1 out of 10. In contrast, only 21.0% (127/552) of participants expressed willingness to voluntarily accept treatment in locked wards, while the majority (425/552, 70.4%) explicitly rejected this option. A binary logistic regression revealed that younger patients were significantly more likely to accept voluntary locked ward treatment (β=−.18, *P*=.04; Figure 1), with each increase in age group reducing the likelihood of voluntary acceptance. In addition, there was a trend suggesting that patients with affective disorders (F30-F39) were less willing to accept voluntary locked ward treatment (β=−.42, *P*=.08). Gender and other diagnostic categories were not significantly associated with willingness to accept locked ward treatment.

### Topic Modeling of Open-Ended Responses

The word counts per response ranged from 1 to 64 words, with a median of 7 (IQR 4-13) and a mean of 9.6 (SD 9.1) words, indicating generally concise participant statements. Regarding term frequency, the top 20 most common words appeared across many documents, reflecting key themes in the dataset. For example, freedom featured in 77 responses, ward in 57, and locked in 41, while other frequent terms included outside (n=24), loss (n=21), patients (n=20), and trapped (n=18; Table 2).

**Table 2.** Top 20 terms by document frequency.

Rank	Term	Document count, n
1	Freedom	77
2	Ward	57
3	Locked	41
4	Like	28
5	Outside	24
6	Loss	21
7	Patients	20
8	Trapped	18
9	Closed	18
10	Imprisoned	16
11	Able	16
12	Prison	14
13	Confined	14
14	Others	14
15	Restricted	13
16	Can	13
17	Freely	12
18	Lack	12
19	Never	11
20	Move	11

**Figure 2.**
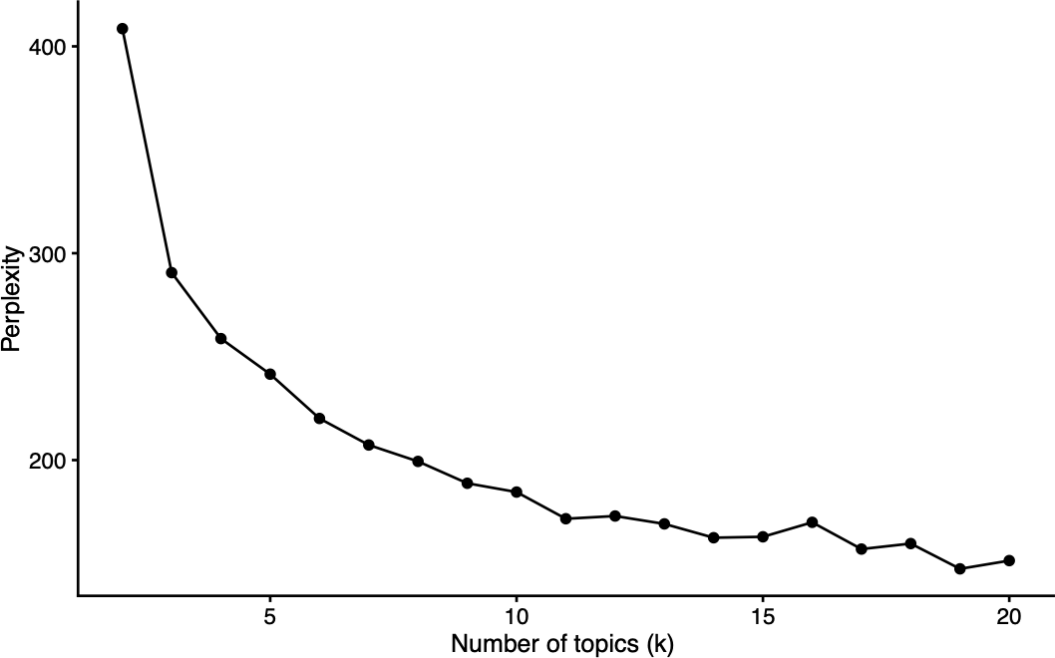
Perplexity scores guiding k-topic selection.

LDA analysis of open-ended survey responses identified 5 latent topics, with model selection guided by perplexity minimization (Figure 2). Thematic analysis of the 5 most salient topics, expressed as probabilistic association of words with topics (β values) derived from the LDA model (Multimedia Appendix 2), revealed nuanced patient perspectives on open and locked ward environments.

**Figure 3.**
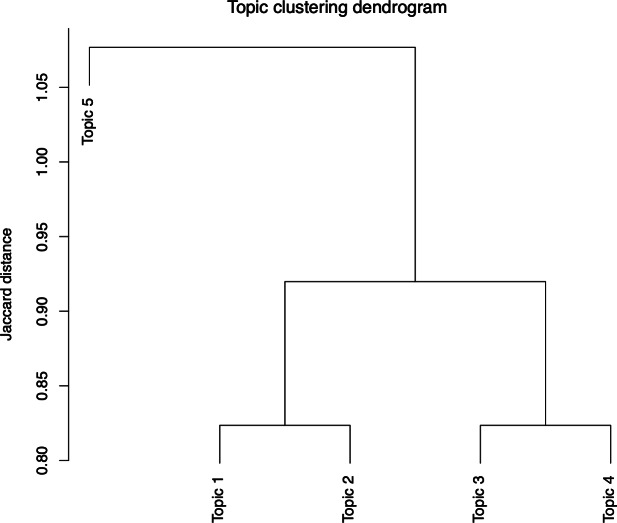
Hierarchical clustering dendrogram of thematic relationships.

**Figure 4.**
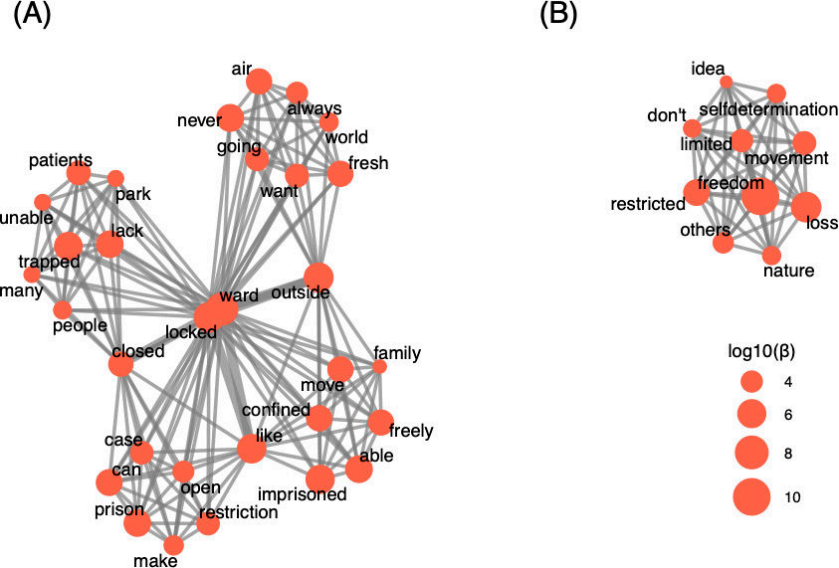
Co-occurrence network based on latent Dirichlet allocation topic analysis. Node size represents log10 of β (the probability of word “w” given topic “k”); edge thickness indicates the frequency of co-occurrence between terms. Node centrality reflects the relative importance of terms within the networks (topics): (A) Restriction and Institutionalization and (B) Autonomy and Self-Determination.

Hierarchical clustering of the topic-word matrix (Figure 3) grouped the 5 dominant themes into 2 clusters, revealing conceptual linkages in patient narratives. Based on the clustering results and the initial thematic interpretation, the optimal number of topics was determined as k=2, identifying 2 overarching themes within the patient narratives (Figure 4).

The final topic analysis revealed the following topics: (1) Restriction and Institutionalization and (2) Autonomy and Self-Determination.

In topic 1, Restriction and Institutionalization (Figure 4A), the recurrence of terms such as “locked,” “trapped,” “confined,” and “imprisoned” suggests an underlying sense of entrapment and loss of control, which can contribute to feelings of helplessness and distress. The rigid structure of the ward, governed by institutional rules limiting movement and requiring permission for basic activities, appears to intensify a perceived lack of agency. This restriction is not only physical but also psychological, reinforcing a sense of stagnation and dependency. The prevalence of these terms suggests a perception of physical and psychological constraint, where institutional rules regulating movement and outdoor access contribute to feelings of disempowerment. The co-occurrence of terms related to “permission” and “restriction” indicates a broader concern regarding autonomy, as patients frequently reference the necessity of approval for routine activities. There is a recurring longing for spending basic time outdoors in the “park,” going for a “walk,” and also a sense of lack of “fresh air.” In addition, terms associated with “isolation” and “separation” highlight the role of limited social contact (friends) in shaping patients’ emotional experiences. The patterns observed suggest that patients often conceptualize their environment as restrictive, with an underlying emphasis on a longing for greater agency and access to the outside world.

Topic 2, Autonomy and Self-Determination (Figure 4B), is anchored by terms such as “freedom,” “loss,” and “movement” and reflects, complementary to topic 1, patients’ concerns about restrictions on personal agency within locked psychiatric wards and their striving toward “freedom,” autonomy, and self-determination. Many respondents articulated a sense of “loss”—not only of physical mobility but also of control over their daily lives. Policies enforcing rigid schedules, such as fixed meal times and supervised activities, were perceived as limiting self-determination. The prominence of “self-determination” in the topic structure underscores the importance patients placed on having an active role in decisions affecting their treatment and daily routines. In addition, the presence of “nature” suggests that access to outdoor environments was seen as integral to autonomy and well-being. Overall, this theme highlights the tension between institutional policies designed for safety and patients’ fundamental need for independence, reinforcing calls for more open, flexible, and participatory care models.

## Discussion

### Principal Findings

The findings of this study underscore the importance of patient perspectives on psychiatric ward policies, with a strong preference for open-door approaches. Locked wards were viewed by many respondents as punitive and counterproductive, contradicting principles of dignity, autonomy, and therapeutic efficacy. A significant source of dissatisfaction was the ward environment itself, with patients frequently reporting that closed units exacerbated stress, isolation, and disempowerment. These concerns were clustered with broader critiques of psychiatric care systems, where physical restrictions were linked to institutional challenges such as rigid hierarchies and limited patient involvement in decision-making. Patients strongly favored open-door environments, associating unrestricted movement with enhanced autonomy, dignity, and engagement in treatment. Conversely, supporters of locked wards emphasized safety concerns, though this perspective was held by a minority. Almost two-thirds of respondents reported that restricted movement increased stress and hindered recovery, particularly among those with prolonged admissions. Notably, perceptions of compromised self-determination reflected a misalignment between institutional priorities and patient needs.

In recent years, the discourse surrounding the reduction of coercive measures in psychiatric settings has gained considerable momentum, particularly in the context of international human rights frameworks. A key impetus for this shift has been the United Nations Convention on the Rights of Persons with Disabilities (CRPD), adopted by the UN General Assembly on December 13, 2006 [45] and ratified by Switzerland on April 15, 2014 [46]. The CRPD affirms that individuals with disabilities are entitled to the full spectrum of human rights and should be enabled to participate equally in social, economic, and public life. Rather than establishing new or separate rights, the Convention reinterprets universal human rights through the lens of disability, emphasizing the need for equal opportunities and protection from discrimination. Within psychiatric care, this paradigm shift has contributed to growing advocacy for “open psychiatry,” an approach that promotes the management of even severely ill patients, including those treated involuntarily, in settings without locked wards [47-49]. This model aims to uphold autonomy and dignity while simultaneously reducing coercion. Our findings are consistent with existing literature, which demonstrates that patient satisfaction and the overall environment in psychiatric settings are significantly improved in open wards, where there is an increased sense of autonomy and enhanced privacy [14,50]. The perceived drawbacks of closed wards are consistent with a qualitative survey of 170 patients, where respondents reported that closed-door environments in psychiatric settings led them to feel “confined,” increased their dependence on staff, and negatively impacted their emotional well-being [51]. Similarly, our findings align with concerns raised by staff, who noted that closed-door environments “make patients feel depressed,” “confined,” and “create additional work” [52]. This survey of acute psychiatry staff also linked locked wards to increased patient aggression, lower treatment satisfaction, and greater symptom severity [52]. While staff responses to open-door policies are less frequently studied, they tend to be more nuanced than the overwhelmingly positive endorsements by patients. In one survey, staff participants described open-door environments as often leading to aggressive conflicts around the exit door [53]. In another survey, some staff members also expressed the belief that a partly locked ward enhanced feelings of safety for patients on the open side. In a separate survey, psychiatrist participants emphasized the necessity of continuous staff presence at the ward entrance as an essential criterion for maintaining an open-door policy. Notably, across all professional groups, there was consensus on the need for increased staffing to facilitate the successful implementation of such policies [15]. Concurrently, findings from another study indicated that professional participants perceived open wards as promoting a sense of freedom and independence for patients. This environment was described as beneficial for therapeutic relationships, fostering increased trust and collaboration between patients and staff [16]. These results underscore the perceived opportunities and challenges associated with open-door policies, emphasizing the need to incorporate staff perspectives. They suggest that implementing open doors without adequate preparation—including appropriate staffing levels and staff training—may inadvertently undermine intended benefits.

Open-door policies require active patient involvement in treatment, with flexibility in strategies to address individual needs. Establishing mutual trust between patients and therapists is essential for effective care. Implementing open-door policies in psychiatric settings may contribute to a more beneficial ward atmosphere and a more patient-centered care model [14,30,50,54,55]. This practice also supports a more professionalized patient assessment [15,56], a reduction in coercive measures [26,30,57,58], a decrease in appeals against patients’ stays [59], and an improvement in psychosocial functioning [60]. Empirical evidence suggests that open-door treatment frameworks are not associated with increased rates of suicide or absconding when compared to more restrictive traditional models and may be accompanied by a reduction in violent incidents [31]. A recent study provided data-driven evidence supporting the efficacy of open-door policies. This pragmatic, randomized controlled, noninferiority trial compared 2 open-door wards with 3 treatment-as-usual acute psychiatric wards at Lovisenberg Diaconal Hospital in Oslo, Norway [29]. The results demonstrated that the open-door policy was noninferior to treatment-as-usual across all outcomes. This pioneering research emphasizes the value of empirical, data-driven approaches to evaluating open-door policies, as opposed to purely ideological perspectives [29]. A systematic review of the types and proportions of antecedents to violence and aggression in psychiatric inpatient settings found that limiting patients’ freedoms, through restrictions or denial of requests, was the most common precursor to incidents, accounting for approximately 25% of all identified antecedents [61]. The reduction of coercive measures and the implementation of an open-door policy in psychiatric units hinge on several critical factors. Avoidance of overcrowding among severely ill patients is paramount, with emphasis placed on protecting the private sphere [62]. Strengthening therapeutic offerings, enhancing patient orientation [54,63], ensuring the approachability of staff [61,62,64], and preserving the personal space and autonomy of patients are integral components. In addition, fostering a positive ward atmosphere and addressing patients’ needs through inclusion are fundamental prerequisites for delivering high-quality care [54,61-65]. The implementation of an open-door policy contributes to a care approach that is more patient-centered and recovery-oriented. This, in turn, leads to a consistent reduction in the use of seclusion and forced medication [30,58]. Open psychiatry is, above all, a matter of institutional and professional attitude, particularly among nursing staff, who play a central role in its implementation. The deliberate decision to forego locked doors fosters closer therapeutic relationships with severely ill patients and necessitates a high degree of creativity, vigilance, and professional commitment to de-escalate potentially volatile situations. Naturally, the successful application of such an approach is contingent upon adequate staffing levels and appropriate personnel resources [50].

Despite compelling international evidence supporting the effectiveness of care models such as Safewards and the implementation of open-door policies in enhancing the inpatient environment and reducing the necessity for containment and restrictive practices [27], a judicious consideration of evidence-based arguments for closed-door policies tailored to specific patient populations remains imperative. Some data suggest that the usage of locked doors aids staff in delivering structured and secure care [66,67], as indicated in a study where advantages identified by patients fell into 6 categories, with 5 of these aligning with responses from the staff. These included protection against external factors, exerting control over patients, facilitating secure and efficient care, and providing safety and relief for significant others or relatives [68]. Furthermore, the rationales for the persistent implementation of door locking may encompass instances involving patients exhibiting suicidal ideation, disorientation, or limited communication skills. Some authors propose that, in certain situations, locking doors may be viewed as less intrusive than intensive observation [25].

While open-door policies are often embraced with optimism, some critics have cautioned against an uncritical endorsement. Coercion and force in psychiatric settings cannot be effectively addressed by categorically denying their legitimacy, universally condemning their use, or viewing patient-initiated violence as inevitable [3]. Rather, acknowledging that, in rare but severe cases, restrictive measures or interventions against a patient’s will may be necessary as a last resort to prevent serious harm enables a more realistic understanding of the core ethical dilemma: in certain situations, it may be impossible to uphold one set of rights without infringing upon another. These scenarios require a careful, context-specific balancing of competing rights and moral principles [3]. When coercive measures are implemented to protect a patient from self-harm against their current natural will, they nonetheless maintain a close and affirmative relationship to the principle of autonomy. Such interventions are only ethically and legally permissible if they align with the patient’s advance directives or presumed will during a period of decisional incapacity. Their objective is to uphold the patient’s reasoned, value-based, and personality-anchored right to self-determination—referred to as “strong autonomy” [69]. The goal of autonomy-focused psychiatry is to make conflicts of rights and interests transparent and to prevent them through proactive measures—limiting coercion and force to an absolute minimum in line with a “least-restrictive” approach. Central to this is the ultima ratio principle, which permits such interventions only when no viable alternatives exist. Each case requires a careful assessment of proportionality and, where feasible, a trial of less restrictive options. If these fail, coercive measures may not only be the last resort but also the ethically and legally appropriate course of action [70].

An unexpected finding emerged in that younger patients, particularly those aged 16‐25 years, were more likely to report willingness to accept treatment in a locked ward setting. This observation contrasts with prior literature suggesting that younger adults tend to exhibit lower levels of trust in physicians [71]. In addition, it appears to diverge from findings indicating that older age is more commonly associated with involuntary psychiatric hospital admissions [72,73]. Several possible explanations may account for this observation. One evidence-based interpretation is that, despite the aversive nature of coercive measures, adolescents and young adults in this age group might nonetheless prioritize the perceived safety and structured environment provided by locked wards [74]. Furthermore, the influence of family and peer pressure tends to play a more substantial role in treatment decisions for younger individuals, as compared to older patients, who are more likely to make autonomous choices regarding their care [75]. Another hypothetical explanation could be that younger patients may have had fewer prior negative experiences with psychiatric hospitalization, including coercion, resulting in a greater willingness to accept such measures. Future research should explore these dynamics in more depth to better understand age-related variations in attitudes toward locked ward treatment.

Despite our study’s compelling findings, there are limitations to consider. The study was conducted at a single-center facility, and the low response rate (19%) may introduce bias, with more severely ill patients potentially underrepresented. While the achieved sample size was robust and the response rate aligned with expectations for this clinical population, the possibility of nonresponse bias remains [39]. It is conceivable that individuals with particularly strong opinions—either favorable or unfavorable—about open-door policies may have been more likely to respond. Conversely, those with less trust in institutional processes or more severe illness-related impairments may have been underrepresented. As such, the views of nonrespondents may differ in meaningful ways, particularly with regard to autonomy, perceived coercion, and satisfaction with care. These limitations should be taken into account when interpreting the generalizability of the findings.

Furthermore, the study did not assess the perspectives of visitors or staff, which could provide additional insights into the effectiveness of open-door policies. The use of text mining techniques, such as LDA, to analyze patient responses presents challenges. LDA, while useful for identifying topic distributions, has limitations in capturing nuanced patient experiences, as it assumes topics are independent and does not account for contextual variation across individuals. The study drew from a stratified random sample of 3163 individuals, ensuring broad representation of patients with diverse experiences and needs. The questionnaire covered multiple relevant domains (person, room, ward, and clinic), allowing for a detailed understanding of patient preferences and needs. The combination of electronic and paper questionnaires maximized participation. Former patient and peer involvement in designing the questionnaire is a strength of this study, as it is crucial for ensuring its relevance and accuracy.

### Conclusions

In conclusion, our study highlights the importance of open-door policies in psychiatric care from the patient perspective, emphasizing autonomy, trust, and engagement in treatment. By aligning institutional practices with patient priorities, psychiatric settings can enhance patient satisfaction and treatment outcomes. Further research is needed to explore the perspectives of staff and visitors, as well as the effectiveness of open-door policies in diverse health care settings.

### Actionable Recommendations

In light of these findings, institutions should consider several practical measures to align with patient preferences and improve ward environments. Ensuring adequate staffing levels and staff training is essential to implement open-door policies safely [15], as it facilitates therapeutic engagement and reduces the need for coercion. Institutions should also prioritize expanding structured therapeutic activities and personalizing treatment plans, fostering a collaborative atmosphere that empowers patients and enhances autonomy. Creating a ward environment that safeguards privacy and preserves personal space is crucial, as it mitigates stress and supports recovery [76]. Moreover, open-door policies should be paired with protections for personal privacy, such as quiet areas, private spaces, and respect for individual boundaries [77,78]. These steps, when combined with a commitment to transparency, inclusivity, and evidence-based practice, can promote a shift toward more patient-centered and recovery-oriented psychiatric care while ensuring patient and staff safety.

## Supplementary material

10.2196/73610 Multimedia Appendix 1 Original questionnaire used in the study.

10.2196/73610 Multimedia Appendix 2 Tabular overview of top terms and corresponding β values for each topic derived from latent Dirichlet allocation analysis.

## Abbreviations

CRPD: Convention on the Rights of Persons with Disabilities
*ICD-10*: *International Statistical Classification of Diseases and Related Health Problems, Tenth Revision*
LDA: latent Dirichlet allocation
UPK: University Psychiatric Clinics
