# Supplementary material for: Patient Perspectives on Open-Door Policies in Psychiatry: Mixed Methods Study
Source: J Med Internet Res. 2025 Aug 8;27:e73610. doi: 10.2196/73610 (PMC12334140; doi:10.2196/73610)
Supplement: Multimedia Appendix 1 [file jmir-v27-e73610-s001.pdf]

Universitäre
Psychiatrische Kliniken
Basel

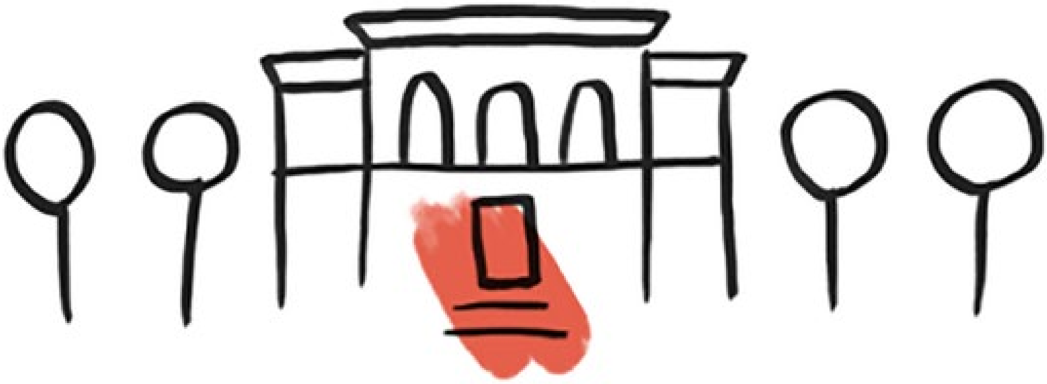

Befragung Neubau Psychiatrie

- Wir bitten Sie für das Ausfüllen der Umfrage einen Kugelschreiber oder ähnliche permanente Schreiber zu benutzen.
- Diese **Umfrage ist anonym** und Ihre Antworten können nicht zu Ihnen persönlich zurückgeführt werden. Sie dürfen also ganz offen antworten. Wir befürworten dies sogar sehr.
- Umfragen können ermüdend sein. Falls Sie nicht alles in einem beantworten können, machen Sie eine Pause und fahren Sie später fort oder lassen Sie für Sie unrelevante Fragen einfach unbeantwortet.

Wir bedanken uns bereits ganz herzlich für Ihre Antworten und **Ihre Mitgestaltung unserer zukünftigen Klinik**.

Sie sind

☐ weiblich ☐ männlich ☐ divers ☐ ohne Angabe

Zu welcher der folgenden Altersgruppen gehören Sie?

☐ 16 – 25 Jahre	☐ 41 – 55 Jahre	☐ 66 – 70 Jahre
☐ 26 – 40 Jahre	☐ 56 – 65 Jahre	☐ über 70 Jahre

Auf welchen stationären Abteilungen der UPK waren Sie bereits?

☐ S4	☐ U1	☐ P1	☐ J
☐ S1	☐ U2	☐ P2	☐ B
☐ C	☐ U3	☐ APA	☐ VTS
☐ S2	☐ VSS	☐ KIS	☐ PTA

Wo verbringen (oder verbrachten) Sie die meiste Zeit während Ihres Klinikaufenthaltes und wieso? *Z.B. im eigenen Zimmer, Aufenthaltsraum, Park oder Raucherzimmer?*

Wo verbringen (oder verbrachten) Sie die wenigste Zeit während Ihres Klinikaufenthaltes und wieso?

A) Patient:innenzimmer

1. Hat Sie an Ihrem Zimmer etwas gestört?

☐ Ja ☐ Nein

<u>Falls ja</u>, was hat Sie an Ihrem Zimmer gestört?

☐ Helligkeit / Beleuchtung
☐ Fehlende Rückzugsmöglichkeit
☐ Raumklima (Hitze / Kälte / Trockenheit)
☐ Raumausstattung (z.B. Möbel, TV, WLAN etc.)
☐ Geräusche / Lärm / Hellhörigkeit / Gerüche
☐ Gebäude / Abteilungszustand
☐ Zu wenig Platz
☐ Mehrbettzimmer
☐ Sonstiges (bitte unten beschreiben):

2. Wie wichtig ist Ihnen die Privatsphäre während Ihres Aufenthalts? Welche Aspekte der Privatsphäre sind Ihnen besonders wichtig?

3. Der Plan im Moment sieht vor, im Neubau ausschliesslich Einzelzimmer zur Verfügung zu stellen. Wie wichtig ist / wäre Ihnen ein Einzelzimmer?
(1-10, 1 = unwichtig, 10 = sehr wichtig)

1	2	3	4	5	6	7	8	9	10
☐	☐	☐	☐	☐	☐	☐	☐	☐	☐

Optionaler Kommentar: ____

____

____

____

____

____

____

____

4. Braucht es in jedem Patient:innenzimmer ein Tablet oder Fernseher?

☐ Ja ☐ Nein

5. Würden Sie gerne Therapien via Tablet / Smartphone / App planen?

☐ Ja ☐ Nein

6. Würden Sie gerne die Möglichkeit zu virtuellen Therapieangeboten nutzen (Apps oder virtuelle Realität)?

☐ Ja ☐ Nein

B) Abteilung

7. Haben Sie zu Ihren Abteilungen Anmerkungen? Was war räumlich <u>gut gelöst?</u>

8. Was war <u>schlecht gelöst</u>?

9. Welche Art von therapeutischen Ressourcen oder Einrichtungen würden Sie gerne auf der Abteilung haben?

- ☐ Fitnessraum
- ☐ Entspannungsbereich
- ☐ Kunst- oder Musiktherapie-räume
- ☐ Gruppentherapieraum
- ☐ Raucherraum
- ☐ Küche
- ☐ Begegnungsraum
- ☐ Sonstige: ______________________________

Sollten diese offen und direkt und jederzeit zugänglich sein?

- ☐ Ja
- ☐ Nein

10. Welche Angebote sollten gestärkt werden und direkt auf einer Abteilung verfügbar sein?

- ☐ Visite
- ☐ Psychotherapie
- ☐ Bezugspflege
- ☐ Sozialarbeit
- ☐ Physiotherapie
- ☐ Ergotherapie
- ☐ Keine
- ☐ Andere: ______________________________

11. Sollten Einzeltherapien und Visiten eher in Ihrem Zimmer (im Falle, dass jede/r Patient/in ein Einzelzimmer hat) oder eher in einem Therapieraum stattfinden?

☐ Im eigenen Zimmer ☐ Im Therapieraum

12. In welcher Grösse der Abteilung würden sie sich am wohlsten fühlen?

☐ 8-12 Patient:innen ☐ 13-16 Patient:innen ☐ 17-20 Patient:innen ☐ spielt keine Rolle

13. Finden Sie es wichtig, dass Abteilungen ein diagnosespezifisches Konzept verfolgen, also dass Patient:innen mit ähnlichen Diagnosen auf einer Abteilung sind?

☐ Finde ich wichtig ☐ Finde ich unwichtig

14. Wie wichtig ist es Ihnen, auf einer offenen Abteilung behandelt zu werden?
(1-10, 1 = unwichtig, 10 = sehr wichtig)

1	2	3	4	5	6	7	8	9	10
☐	☐	☐	☐	☐	☐	☐	☐	☐	☐

15. Würden Sie freiwillig auch auf einer geschlossenen Abteilung behandelt werden wollen?

☐ Ja ☐ Nein

16. Falls nein: Was sind die Nachteile einer geschlossenen Abteilung?

17. Finden Sie es wichtig, dass ältere Menschen (über 65 Jahre) auf eigenen Schwerpunktabteilungen getrennt behandelt werden können oder sollten auf Abteilungen ältere Menschen integrativ behandelt werden?

☐ Ältere Menschen getrennt ☐ Integrativ

18. Gibt es weitere Anforderungen oder Bedürfnisse hinsichtlich der Grösse oder Aufteilung der Abteilungen in der Klinik?

19. Würden Sie lieber auf der Abteilung essen oder in einem gemeinsamen Restaurant (mit Buffet) mit anderen Abteilungen und Mitarbeitenden zusammen?

☐ Abteilung ☐ Restaurant

Optionaler Kommentar:

20. Wie wichtig ist Ihnen die Verfügbarkeit einer direkten Ansprechperson (im Sinne einer offenen Rezeption) für alltägliche Anliegen und Fragen?
(1-10, 1 = unwichtig, 10 = sehr wichtig)

1	2	3	4	5	6	7	8	9	10
☐	☐	☐	☐	☐	☐	☐	☐	☐	☐

21. Muss diese Person eine Pflegefachperson sein oder könnte das auch eine administrative Fachkraft (Rezeption, Hotellerie etc.) sein?

☐ Pflegefachperson direkt ansprechbar ☐ Rezeption direkt ansprechbar

C) Umgebung / Klinikareal

22. Wie wichtig ist Ihnen die Umgebung, der Park?
(1-10, 1 = unwichtig, 10 = sehr wichtig)

1	2	3	4	5	6	7	8	9	10
☐	☐	☐	☐	☐	☐	☐	☐	☐	☐

23. Wie wichtig ist Ihnen ein Zugang zu einem Aussenbereich wie einem Garten oder einem Innenhof?
(1-10, 1 = unwichtig, 10 = sehr wichtig)

1	2	3	4	5	6	7	8	9	10
☐	☐	☐	☐	☐	☐	☐	☐	☐	☐

24. Muss dieser Zugang direkt von der Abteilung ebenerdig möglich sein oder kann er auch über einen Lift erfolgen?

☐ Ebenerdige Abteilung ☐ Zugang über eine Treppe / Lift

25. Welche Art von Räumen oder Einrichtungen sind Ihnen während Ihres Aufenthalts in einer psychiatrischen Klinik besonders wichtig?

☐ Fitnessraum
☐ Küche
☐ Fernsehzimmer
☐ Cafeteria
☐ Eigenes Zimmer
☐ Gemeinschafts- / Begegnungsraum
☐ Kunstatelier
☐ Raucherzimmer
☐ Garten
☐ Musikraum / Musikinstrumente
☐ Sportplatz

26. Hat Sie an der räumlichen Situation an den UPK (Klinikareal, gewisse Räume oder Umgebung) etwas gestört?

D) Behandlung / Angebot

27. Wie wichtig ist Ihnen die Möglichkeit zu einer…
(1-10, 1 = unwichtig, 10 = sehr wichtig)

…vollstationären Behandlung?
Werktags und am Wochenende im Spital mit der Möglichkeit für Ausgang und Probeübernachtungen

1	2	3	4	5	6	7	8	9	10
☐	☐	☐	☐	☐	☐	☐	☐	☐	☐

…tagesklinischen Behandlung?
Werktags tagsüber im Spital mit Übernachtung und Wochenende zu Hause

1	2	3	4	5	6	7	8	9	10
☐	☐	☐	☐	☐	☐	☐	☐	☐	☐

…Behandlung in einer Nachtklinik?
Aufenthalt in der Klinik Abends und Nachts, tagsüber nicht im Spital

1	2	3	4	5	6	7	8	9	10
☐	☐	☐	☐	☐	☐	☐	☐	☐	☐

…Home Treatment?
Therapeut:innen kommen zu Ihnen nach Hause, kein Spitalaufenthalt

1	2	3	4	5	6	7	8	9	10
☐	☐	☐	☐	☐	☐	☐	☐	☐	☐

…ambulanten Behandlung?
Sie besuchen Ihre Therapeutin oder Ihren Therapeuten in der Klinik

1	2	3	4	5	6	7	8	9	10
☐	☐	☐	☐	☐	☐	☐	☐	☐	☐

…ambulante Behandlung via Telemedizin (zum Beispiel via ZOOM)?
Sie interagieren zuhause auf Ihrem Computer mit der ambulanten Therapeutin oder dem ambulanten Therapeuten

1	2	3	4	5	6	7	8	9	10
☐	☐	☐	☐	☐	☐	☐	☐	☐	☐

28. Wenn Sie uneingeschränkt wählen könnten: Welche Art der Behandlung würden Sie bevorzugen?

☐ Stationär	☐ Home Treatment	☐ Nachtklinik
☐ Tagesklinisch	☐ Ambulant	☐ Telemedizin

29. Wie wichtig ist es Ihnen, dass Sie vom gleichen Team stationär, tagesklinisch und ambulant behandelt werden?
(1-10, 1 = unwichtig, 10 = sehr wichtig)

1	2	3	4	5	6	7	8	9	10
☐	☐	☐	☐	☐	☐	☐	☐	☐	☐

30. Finden Sie, dass ambulante Sprechstunden in räumlicher Nähe zu den jeweiligen stationären Einheiten angeboten werden sollten oder sollten diese lieber extern in einem anderen Gebäude stattfinden?

☐ In räumlicher Nähe ☐ In einem anderen Gebäude

31. Haben Sie weitere Anregungen oder Vorschläge, die Sie bezüglich des Neubaus einer Psychiatrie teilen möchten?

32. Würden Sie tendenziell in Zukunft eher in den bestehenden Abteilungen behandelt werden wollen oder in einem Neubau?

☐ Bestehende Abteilungen ☐ Neubau

Bitte legen Sie den ausgefüllten Fragebogen ins vorfrankierte Rückantwortkuvert und senden Sie den Fragebogen damit ans *Institut für Evaluationsforschung Basel*, *Postfach 4477*, *4002 Basel.*

Vielen Dank für Ihre Teilnahme an dieser Umfrage!

Ihre Meinung ist uns wichtig, um die Zukunft der Psychiatrie bestmöglich an die Bedürfnisse der Patient:innen anzupassen.
